# Estimates of hospitalization attributable to influenza and RSV in the US during 1997–2009, by age and risk status

**DOI:** 10.1186/s12889-017-4177-z

**Published:** 2017-03-21

**Authors:** Gonçalo Matias, Robert Taylor, François Haguinet, Cynthia Schuck-Paim, Roger Lustig, Vivek Shinde

**Affiliations:** 1 GSK, Wavre, Belgium; 2 Sage Analytica, Bethesda, MD USA; 3 GSK, King of Prussia, USA; 4 Present Address: Novavax Vaccines, Washington, DC USA

**Keywords:** A/H3N2, Influenza, Mortality, Respiratory broad, Respiratory syncytial virus

## Abstract

**Background:**

Estimates of influenza and respiratory syncytial virus (RSV) burden must be periodically updated to inform public health strategies. We estimated seasonal influenza- and RSV-attributable hospitalizations in the US from 1997 to 2009 according to age and risk status (NCT01599390).

**Methods:**

Multiple linear regression modelling was used to attribute hospitalizations to influenza or RSV using virological surveillance and hospitalization data. Hospitalization data were obtained from the US Nationwide Inpatient Sample and virology data were obtained from FluView (Centers for Disease Control and Prevention). Outcomes included any mention of ICD-coded respiratory disease and cardiorespiratory disease diagnoses. We also explored a broader definition of respiratory disease that included mention of relevant respiratory sign/symptoms and viral infection (“respiratory broad”).

**Results:**

Applying the respiratory broad outcome, our model attributed ~300,000 and ~200,000 hospitalizations to influenza and RSV, respectively. Influenza A/H3N2 was the predominant cause of influenza-related hospitalizations in most seasons, except in three seasons when influenza B was dominant; likewise, A/H3N2 caused most influenza-related hospitalizations in all age segments, except in children <18 years where the relative contribution of A/H3N2 and B was similar. Most influenza A- and B-related hospitalizations occurred in seniors while approximately one half and one third of all RSV-related events occurred in children 0–4 years and seniors 65+ years, respectively. High-risk status was associated with higher risk of both influenza- and RSV-attributable hospitalizations in adults, but not in children.

**Conclusions:**

Our study assessed the burden of influenza and RSV, information that is important for both cost effectiveness studies and for prioritization of the development of antivirals and vaccines. For seniors, we found that the burdens of influenza and RSV were both substantial. Among children <18 years, about half of all influenza hospitalizations were due to influenza B, most occurring in children without noted risk conditions. RSV hospitalizations among children were confined to those 0–4 years. Our study also demonstrated the importance of the outcome used to estimate hospitalization burden. Our findings highlight the burden of influenza among children regardless of risk status and underscore the prevalence of RSV infections among both young children and older adults.

**Electronic supplementary material:**

The online version of this article (doi:10.1186/s12889-017-4177-z) contains supplementary material, which is available to authorized users.

## Background

Viral respiratory illnesses such as influenza and respiratory syncytial virus (RSV) impose a substantial burden of hospitalization [1–5]. Influenza has a disproportionate impact on the young, older adults, and persons with underlying high risk medical conditions [1, 2]. Clinical manifestations of influenza virus infection range from asymptomatic or mild upper respiratory illness to severe lower respiratory tract disease or exacerbation of chronic respiratory and cardiac disease. The clinical impact of influenza is not confined to respiratory conditions. In young children in particular, the clinical presentation of influenza can be diverse, and may include gastrointestinal symptoms and febrile convulsions [6]. In adults, excess cardiovascular outcomes have been attributed to influenza [7]. The burden of RSV in young children has long been recognized [3–5], and more recent studies have also identified a substantial burden in older adults [8–10]. Clinical manifestations of RSV infection range from sinusitis and otitis media to bronchiolitis and pneumonia.

Because the burden of seasonal influenza hospitalizations is difficult to assess directly, historically it has been estimated by statistically modeling excess wintertime health-related outcomes over the background rates of the same outcomes recorded outside the wintertime period [11]. The US Centers for Disease Control and Prevention (CDC) has applied a variety of statistical modeling techniques over the past decades to derive these estimates. More recently, virological surveillance-guided multiple regression models that include International Classification of Disease (ICD)-coded outcomes data as additional model variables have been used to derive pathogen-specific estimates of disease burden, including the burden of RSV. The primary advantage of this approach is that the specific and concomitant burden of disease associated with multiple pathogens is estimated while controlling for other disease drivers [12–14].

The burden of influenza varies from season to season and from decade to decade, both overall and in its impact on different age groups, depending in part on the dominant virus type or subtypes in circulation [15]. Influenza A/H1N1 and A/H3N2 viruses have co-circulated for the past three decades; although A/H3N2 has caused most influenza A illness over this period, A/H1N1 has predominated periodically [16]. Two distinct lineages of the influenza B virus, Victoria and Yamagata, have co-circulated since at least 1983. In the 1990s, the Victoria lineage was not detected in North America, but re-emerged during 2001 [17]. Both lineages have subsequently co-circulated or alternated. Suboptimal vaccine protection might occur in seasons where the predominant influenza B virus is from the lineage that is not included in the trivalent seasonal influenza vaccine [18, 19].

To inform vaccination strategies and to prompt accelerated development of novel vaccine technologies, it is critical to thoroughly characterize evolving epidemiological patterns of virus circulation and the burden of viral illness. In the present study, we estimated the burden of influenza- and RSV-associated hospitalizations in the US between 1997 and 2009. The study had multiple aims. Firstly, it evaluated the relative burden of influenza versus RSV. Secondly, it evaluated the relative burden of influenza A versus influenza B, which is important in assessing the value of the quadrivalent influenza vaccine. Thirdly, it evaluated the burden of illness by age and risk status. Individuals at high risk, including those who are elderly, immunocompromised and chronically ill, generally experience higher rates of severe outcomes from viral infections and are usually prioritized for vaccination [20]. Finally, to better estimate the burden of influenza and RSV disease, the study used an outcome introduced in a related study of influenza- and RSV-associated mortality [21], “respiratory disease broadly defined” (“respiratory broad”), which may provide a better balance of sensitivity and specificity compared with estimates derived using classical definitions.

## Methods

The objective of this retrospective database analysis (NCT01599390) was to estimate the numbers and rates of hospitalization attributable to influenza and RSV in the US, stratified by age and risk status, for 12 winter seasons. Total influenza- and RSV-attributable hospitalization and the relative contribution of the different influenza types and subtypes were assessed. The analytical approach, as previously described [21], used multiple linear regression modelling of time series data extracted from multiple healthcare and virus surveillance databases. The analysis used several definitions of influenza- and RSV-related clinical outcomes, and the model attributed a proportion of each outcome to either influenza or RSV.

### Study population and hospitalization data

The hospitalization data source was the US Nationwide Inpatient Sample (NIS). All subjects of any age who were recorded in the NIS during the period 1st October 1997 to 31st March 2009 were included. Data recorded after 31st March 2009 were excluded to avoid bias associated with the circulation of the 2009 A/H1N1 pandemic influenza virus. Data included demographic, geographic, and ICD-coded information including discharge diagnosis and co-morbid conditions. Records were excluded if they had data missing from the age, primary discharge diagnosis, or admission month fields. Hospitalizations were classified according to ICD codes (Table 1). Informed consent was not required as this was an anonymized database study.

**Table 1:** Definition of hospitalization outcomes expressed in ICD9 codes

Outcome	ICD9 codes
Pneumonia and influenza	480-488
Respiratory broad	460-519, 079, 786.0-786.4, 786.7-786.9
Respiratory	460-519
Cardiorespiratory disease	390-519
Sepsis	038, 771.81

*ICD* International Classification of Diseases

At the time of the study, the NIS data source consisted of all discharge records from a 20% sample of US hospitals, chosen to be representative of the US as a whole and four US regions. Details of the NIS are available at http://www.hcup-us.ahrq.gov/nisoverview.jsp. Monthly counts of hospitalizations were extracted, stratified by age (0-4, 5-17, 18-49, 50-64, 65-74, 75+ years), region, discharge status (dead or alive), and risk status (high or low). The risk status of each subject was determined from co-morbidity diagnosis codes listed. Persons were considered at high risk if any of the following underlying medical conditions known to increase risk of complications of influenza were mentioned: chronic obstructive pulmonary disease, cardiovascular disorders, kidney disorders, diabetes, immunosuppression, liver disorders, stroke, and central nervous system disorders. Denominator data for age-risk strata were incorporated into the study database, and incidence rates were determined for each outcome by 100,000 population. To calculate rates for the high risk and low risk populations, data from the National Health Information Survey (NHIS) was used. These data have been collected by the US Census Bureau through personal household interviews for many years. The proportions of persons answering “yes” to any condition corresponding to the study’s high risk definitions provided weights used to estimate the prevalence of the high risk status in the population.

Compliance with relevant legal requirements for data privacy was ensured through the anonymization and de-identification of the data. Specifically, the patient information in the NIS data contained no personally identifiable information. As it was not possible to connect any data with any personally identifiable information, no patient informed consent was required. Similarly, ethics approval was not sought for this retrospective database study of anonymized data.

### Definition of outcomes

The study included a variety of hospitalization outcomes, ranging from narrow to broad. Five outcomes are reported in this paper: (i) pneumonia and influenza (P&I); (ii) a novel outcome referred to as respiratory disease broadly defined which includes respiratory diseases, cough, breathing abnormalities, fever, and viral infections not otherwise specified (hereafter referred to as respiratory broad); (iii) respiratory; (iv) cardiorespiratory; (v) sepsis (Table 1). In the NIS, both primary and secondary diagnoses are registered. The respiratory codes included all respiratory illnesses, while the cardiorespiratory codes also included all cardiovascular disorders; a complete list can be found at http://icd9cm.chrisendres.com. For all outcomes, two analyses were conducted, one including cases with any mention of the relevant ICD codes as a primary or secondary diagnosis and the other including only cases with the relevant ICD codes mentioned as the primary diagnosis.

### Virology data

The virology data source was the weekly influenza update, FluView, published by the CDC [22]. The weekly numbers of respiratory samples that were laboratory-confirmed as positive for influenza A and B types and subtypes in each of the four US statistical census regions were obtained. These data represent combined reporting to the CDC of approximately 85 World Health Organization (WHO) collaborating laboratories and 60 National Respiratory and Enteric Virus Surveillance System (NREVSS) laboratories. Data were collected between week 40 of the first calendar year of a season and up to and including week 20 of the second calendar year of the season. Weekly RSV surveillance data (counts of laboratory-confirmed RSV viruses, from each region and nationally each week) were obtained from the NREVSS [23]. Age-specific virological data were not available.

### Statistical analysis

#### Preparation of time series data

The statistical modelling approach to attribute ‘excess hospitalizations’ using national time series has only been expanded to include RSV in the early 2000s [12]. Weekly influenza and RSV virology time series were constructed by dividing the weekly total of influenza (according to type and subtype) or RSV positive samples by the seasonal total of influenza or RSV tests. Hospitalization data were available only by month, whilst virus surveillance data were available by week. To account for this, weekly values of aggregate hospitalization outcome counts were estimated by interpolation (SAS ‘Proc Expand’ with spline). Each week’s virus surveillance data were appended to the corresponding week’s hospitalization outcome estimates. Given that presentation with influenza can occur some time after the onset of infection [15], we assessed whether lagging the virological data by 1–3 weeks would improve model fit. As model fit was not improved, we did not introduce a lag.

Exploratory data analysis revealed that the proportion of admissions in the weighted NIS sample falling into a particular age group in a particular year could vary substantially. This occurred because hospitals joined and/or left the NIS data supply effort, the fact that catchment populations of hospitals had varying age profiles, and the lack of age-stratification in the NIS weighting scheme. This presented only a minor problem for common outcomes. However, the scarcer an outcome was within an age group (e.g. “cardiovascular” in younger groups), the more likely it was that year to year fluctuation would cause problems in modeling. The weighted percentage of hospital admissions (all-cause) in each age group within each region for each year was compared with the annual total population of each age group within the corresponding region. The NIS weights were then adjusted so that the annual changes in hospital admissions in each region and age group corresponded to the annual changes in the corresponding population. For example, if the proportion of the population in a particular age group and particular region was 20% in 1998 and 20.5% in 1999, and the proportion of hospital admissions in the same age group and region was 10% in 1998 and 11% in 1999, then the population share for that age group and region expanded by 2.5% over the year, whilst the hospital admissions share increased by 10%. Observing that, for the most part, admissions trends within an age group and region did tend to track those in the population, the admissions for each year were reweighted within each region and age group so that, within a region, the change in admissions share for an age group corresponded to the change in population share for that age group. The result was a partial smoothing of the annual total admissions series for the outcomes analyzed. For most age groups, regions and year-to-year transitions, the reweighting was minimal.

#### Regression model

The analysis, similar to our previously published approach for estimating influenza mortality [21], was performed using SAS version 9.3. For each outcome, census region, age, and risk stratum, the same multiple linear regression model form was applied. The model form used is given by the equation:

$$ \begin{array}{c}\hfill \begin{array}{l}\mathrm{Y} = {\upbeta}_1 + {\upbeta}_2*\left(\mathrm{RSV}\right) + {\upbeta}_3*\left(\mathrm{Influenza}\ \mathrm{A}/\mathrm{H}1\right) + {\upbeta}_4*\left(\mathrm{Influenza}\ \mathrm{A}/\mathrm{H}3\right) + {\upbeta}_5*\left(\mathrm{Influenza}\ \mathrm{B}\right) + {\upbeta}_6*\  \sin \\ {}\left(2\uppi \mathrm{t}/52.18\right) + {\upbeta}_7* \cos\ \left(2\uppi \mathrm{t}/52.18\right) + {\upbeta}_8*\mathrm{t} + {\upbeta}_9*{\mathrm{t}}^2 + {\upbeta}_{10}*{\mathrm{t}}^3\kern0.5em \end{array}\hfill \\ {}\hfill \hfill \end{array} $$

where Y is the incidence (rate) of outcome for each time period t, RSV and influenza are the proportion of laboratory isolates during t (week), sin and cos are harmonic functions of t (week), and the remaining terms track other types of secular trends in the data. The time period t used in the model is a running index of weeks starting 1^st^ October 1997 and ending 31^st^ March 2009. The RSV term controls for the effect of concurrent RSV epidemics. The relative goodness of fit was used to identify the best model form across all age groups by selecting the form with the highest adjusted R^2^; this model form was then applied across all strata. We found a substantial improvement in the adjusted R^2^ value when introducing the virology terms into the base secular model. Visual inspection showed that spikes during the winter-seasonal period are captured well by the model.

Incidence rates for each outcome per 100,000 population were calculated using census data. National Health Information Survey data were used to provide estimates of the prevalence of high risk status in the general population. For each regional seasonal burden estimate and for each influenza type/subtype and for RSV, each weekly point estimate was multiplied by the regression coefficient, then the weekly estimates were aggregated over the entire season.

The overall national burden attributable to each pathogen was determined by aggregating all positive regional point estimates within each stratum by adding the counts and converting to rates. Negative burden estimates were not included in the aggregations as they are not biologically plausible.

## Results

### Model fit

The model fit was generally good in older age groups and in young children aged 0–4 years. The adjusted R^2^ ranged for the respiratory broad outcome from a high of 0.90 in the 75+ years age group to a low of 0.36 in the 5-17 years age group, which is the group with the smallest numbers of hospitalizations and thus the most likely to show a lower fit. In all cases, the addition of the virology terms to the model increased R^2^, and usually by a substantial amount. For example, for the respiratory broad outcome, adding the virology terms increased the adjusted R^2^ by 7% to 70%. Results were similar for the cardiorespiratory outcome, and even better for the P&I outcome, with R^2^ ranging from 0.67 to 0.93 across age groups.

### Estimates of hospitalization burden using various outcomes

#### Outcomes with the relevant ICD codes among any of the discharge diagnoses (“any mention”)

Over the 12 winter seasons during 1997–2009, the model estimated an average seasonal burden of 297,548 (range 123,077–473,027) influenza-attributable hospitalizations when applied to time series using the respiratory broad outcome (any mention); this is equivalent to a mean annual rate of 102 per 100,000 population (Table 2). The highest number of influenza-attributable hospitalizations was associated with influenza A/H3N2 (184,995 [3750–392,352]; 64 per 100,000 population), followed by influenza B (88,775 [2882–188,309]; 30 per 100,000 population) and influenza A/H1N1 (23,778 [49–77,994]; 8 per 100,000 population). The hospitalization burden of RSV was also high, although lower than the burden of influenza: 186,155 (151,326–218,476) hospitalizations, equivalent to an annual mean rate of 64 per 100,000 population (Table 2). As expected, the number of influenza- and RSV-attributable hospitalizations varied depending upon the outcome used (Table 2).

**Table 2:** Average seasonal burden of hospitalization attributable to influenza and RSV in the US, 1997–2009

	Influenza								RSV	
	A/H1N1		A/H3N2		B		Total		Total	
	Number(SD, range)	Rate^a^ (SD, range)	Number(SD, range)	Rate^a^(SD, range)	Number(SD, range)	Rate^a^ (SD, range)	Number(SD, range)	Rate^a^ (SD, range)	Number (SD, range)	Rate^a^(SD, range)
Any mention (primary and secondary diagnoses)										
Pneumonia and influenza	5575 (6413, 10-17224)	2 (2, 0-6)	109605 (73305, 2180-226479)	38 (25, 1-77)	36044 (25264, 1197-76387)	12 (9, 0-25)	151224 (60184, 42931-231692)	52 (21, 14-78)	83826 (9886, 68685-99838)	29 (4, 23-37)
Respiratory broad	23778 (27583, 49-77994)	8 (9, 0-26)	184995 (125020, 3750-392352)	64 (43, 1-134)	88775 (62416, 2882-188309)	30 (21, 1-62)	297548 (95106, 123077-473027)	102 (32, 40-156)	186155 (20695, 151326-218476)	64 (9, 51-81)
Respiratory	22701 (26366, 47-74814)	8 (9, 0-25)	178546 (120620, 3607-378232)	62 (42, 1-129)	84185 (59163, 2754-178530)	29 (20, 1-59)	285432 (91614, 117032-452653)	98 (30, 38-149)	181200 (20786, 146674-214110)	63 (9, 50-79)
Cardiorespiratory	63731 (74696, 128-212553)	22 (25, 0-71)	173091 (119477, 3570-374648)	60 (41, 1-128)	113390 (79442, 3723-232055)	39 (27, 1-76)	350212 (90982, 210388-551170)	120 (29, 69-181)	226017 (25505, 183666-260765)	78 (10, 62-96)
Sepsis	3071 (3632, 6-10653)	1 (1, 0-4)	7336 (4808, 143-14870)	3 (2, 0-5)	6152 (4456, 205-14955)	2 (1, 0-5)	16559 (5924, 9799-31729)	6 (2, 3-10)	7514 (1796, 6105-11918)	3 (1, 2-4)
Primary diagnosis										
Pneumonia and influenza	3322 (3832, 6-9965)	1 (1, 0-3)	75032 (51807, 1617-153996)	26 (18, 1-53)	21270 (15053, 830-42436)	7 (5, 0-15)	99624 (41556, 20428-155443)	35 (15, 7-53)	56790 (10033, 46316-77900)	20 (4, 16-29)
Respiratory broad	7176 (8166, 17-20050)	2 (3, 0-7)	134641 (91445, 2859-276542)	47 (32, 1-94)	48978 (34449, 1775-94178)	17 (12, 1-33)	190795 (71208, 52036-279807)	66 (25, 17-96)	152034 (23947, 122280-193118)	53 (10, 41-71)
Respiratory	6712 (7632, 15-18922)	2 (3, 0-6)	131188 (89045, 2778-269087)	46 (31, 1-92)	47105 (33111, 1711-90828)	16 (11, 1-32)	185005 (69695, 50038-272219)	64 (24, 16-93)	149307 (23197, 119731-188735)	52 (10, 41-70)
Cardiorespiratory	30603 (35872, 59-97842)	10 (12, 0-32)	140248 (97272, 3049-292807)	49 (34, 1-100)	59983 (42372, 2196-118559)	21 (15, 1-42)	230834 (58467, 94469-310270)	80 (20, 31-102)	142301 (23945, 111992-184119)	49 (10, 38-68)
Sepsis	1075 (1316, 2-3824)	0 (0, 0-1)	2692 (1726, 47-4949)	1 (1, 0-2)	3537 (2873, 106-10486)	1 (1, 0-3)	7304 (3540, 3471-17020)	2 (1, 1-6)	2526 (877, 1716-4614)	1 (0, 1-2)

*SD* interseasonal standard deviation, *RSV* respiratory syncytial virus

^a^Annual mean rate per 100,000 population

#### Outcomes with the relevant ICD codes as the primary diagnosis

As expected, the model estimated fewer influenza- and RSV-attributable hospitalizations using the relevant ICD codes mentioned specifically as the primary diagnosis. For influenza, the pattern of hospitalization for the primary diagnosis was similar to that of any mention: the highest number of hospitalizations was associated with the cardiorespiratory outcome (230,834 [range 94,469–310,270]), followed by respiratory broad (190,795 [52,036–279,807]), respiratory (185,005 [50,038–272,219]), and P&I (99,624 [20,428–155,443]) (Table 2). The number of RSV-attributable hospitalizations was lowest for the P&I outcome (56,790 [46,316–77,900]), but was similar for the cardiorespiratory (142,301 [111,992–184,119]), respiratory broad (152,034 [122,280–193,118]) and respiratory outcomes (149,307 [119,731–188,735]). Influenza- and RSV-attributable hospitalizations for the sepsis (primary) outcome were 7304 (3471–17,020) and 2526 (1716–4614), respectively (Table 2).

### Seasonal variation in hospitalization burden

**Fig. 1:**
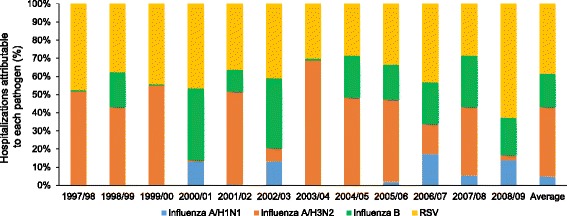
Proportion of total hospitalizations attributable to influenza and RSV by season in the US, 1997–2009 (respiratory broad outcome, any mention)

The total number of influenza-attributable hospitalizations with the respiratory broad outcome (any mention) varied considerably across the 12 seasons covered by the model (Additional file 1: Table S1). In contrast, annual numbers of RSV-attributable hospitalizations varied less over the same period. Nonetheless, in an average season, influenza accounted for 62% of all influenza- and RSV-attributable hospitalizations (Fig. 1).

Although influenza A/H3N2 was predominant in most seasons, influenza B was predominant in three of the 12 seasons, and was associated with 74% of influenza-attributable hospitalizations in 2000/01, 65% in 2002/03, and 56% in 2008/09 (Fig. 1; Additional file 1: Table S1). In a further five seasons (1998/99, 2004/05, 2005/06, 2006/07, 2007/08), influenza B was responsible for approximately 30–40% of influenza-attributable hospitalizations (Fig. 1; Additional file 1: Table S1).

### Age-specific estimates of hospitalization burden

**Fig. 2:**
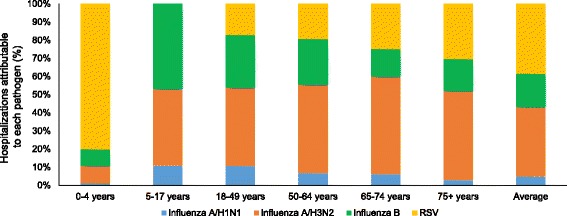
Proportion of total hospitalizations attributable to influenza and RSV by age in the US, 1997–2009 (respiratory broad outcome, any mention)

The average seasonal burden of influenza-attributable hospitalizations meeting the respiratory broad (any mention) outcome varied across age groups (Table 3), with approximately one half occurring in adults over 65 years of age, and one third in adults over 75 years of age. The number of hospitalizations was lower in children, with the 0-4 and 5–17 year age groups accounting for approximately 8% and 4% of the overall burden, respectively. Influenza A was predominant in all age groups, although the distribution of influenza A and B hospitalizations was about 1:1 in both pediatric age groups. In contrast, in seniors 75+ years of age, the ratio was approximately 4:1, consistent with a far higher impact of influenza A/H3N2 in older adults (Table 3 and Fig. 2). Influenza A/H1N1 was responsible for the fewest attributable hospitalizations in all age groups (Fig. 2).

**Table 3:** Average seasonal burden of hospitalization attributable to influenza and RSV according to age in the US, 1997–2009 (respiratory broad outcome, any mention)

	Influenza								RSV	
	A/H1N1		A/H3N2		B		Total		Total	
	Number(SD, range)	Rate^a^ (SD, range)	Number(SD, range)	Rate^a^(SD, range)	Number(SD, range)	Rate^a^ (SD, range)	Number(SD, range)	Rate^a^ (SD, range)	Number(SD, range)	Rate^a^ (SD, range)
0-4 years	1563 (1766, 6-4716)	8 (9, 0-24)	12087 (8427, 234-25121)	62 (43, 1-127)	11496 (8145, 458-23327)	58 (42, 2-119)	25146 (7090, 10522-35031)	128 (36, 52-173)	100618 (14273, 83191-129041)	514 (84, 418-680)
5-17 years	1195 (1382, 2-3815)	2 (3, 0-7)	4466 (3035, 87-9326)	8 (6, 0-17)	4989 (3492, 169-10091)	9 (7, 0-19)	10650 (3316, 5891-18054)	20 (6,12-33)	0 (0, 0-0)	0 (0, 0-0)
18-49 years	7149 (8266, 16-22536)	5 (6, 0-17)	28560 (19488, 569-61736)	21 (15, 0-46)	19172 (13492, 622-41023)	14 (10, 0-30)	54881 (16061, 30529-92199)	41 (12, 23-68)	11365 (1924, 9274-15817)	9 (1, 7-12)
50-64 years	4860 (5655, 9-16319)	10 (11, 0-30)	34042 (23554, 649-75129)	72 (49, 1-154)	17510 (12600, 479-40661)	35 (25, 1-73)	56411 (20851, 26748-101493)	117 (36, 47-182)	13441 (2911, 10902-20093)	28 (4, 22-36)
65-74 years	4252 (5075, 7-15196)	22 (26, 0-77)	34495 (23184, 701-71337)	183 (124, 4-382)	9712 (6876, 303-21581)	51 (36, 2-105)	48459 (17474, 17282-74053)	256 (91, 83-385)	15997 (2670, 12009-21427)	84 (12, 64-103)
75+ years	4760 (5669, 9-16809)	27 (32, 0-93)	71345 (47891, 1510-149703)	414 (279, 9-854)	25896 (18344, 757-56326)	148 (104, 5-308)	102001 (38054, 31679-152199)	589 (216, 173-864)	44734 (5677, 35770-56304)	258 (33, 201-307)
All ages	23778 (27583, 49-77994)	8 (9, 0-26)	184995 (125020, 3750-392352)	64 (43, 1-134)	88775 (62416, 2882-188309)	30 (21, 1-62)	297548 (95106, 123077-473027)	102 (32, 40-156)	186155 (20695, 151326-218476)	64 (9, 51-81)

*SD* interseasonal standard deviation, *RSV* respiratory syncytial virus

^a^Annual mean rate per 100,000 population

In contrast, approximately one half of all RSV-attributable hospitalizations occurred in children aged 0–4 years and approximately one third in adults aged 65+ years, while no RSV-attributable hospitalizations occurred in children aged 5–17 years (Table 3; Fig. 2). The RSV burden in seniors over 65 years was substantial, accounting for more than 60,000 hospitalizations in an average year (Table 3; Fig. 2), and with far less seasonal variability than influenza (data not shown).

Compared with RSV, influenza (all subtypes) accounted for a higher proportion of hospitalizations in all age groups, except children 0–4 years of age, where RSV accounted for 80% of hospitalizations identified by the respiratory broad outcome (Fig. 2). Influenza was responsible for approximately five times as many hospitalizations as RSV in the 18–49 year age group, four times as many in the 50–64 year age group, three times as many in the 65–74 year age group, and twice as many in the 75+ year age group (Table 3).

### Differences in hospitalization burden according to risk status

Overall, high risk status was associated with higher numbers of influenza-attributable hospitalizations. Applying the respiratory broad outcome (any mention), the relative hospitalization rate for high versus low risk status was 7.7 (Table 4). However, the pattern was not consistent between age groups. In children, high risk status was associated with fewer influenza-attributable hospitalizations compared with low risk status. The relative rate of influenza-attributable hospitalizations in high versus low risk adults increased with age, peaking at age 50–64 years with a rate ratio of 8.9, followed by a decline to 7.2 in age 65–74 years and further to 4.9 in age 75+ years (Table 4). Similar findings were observed for the number of hospitalizations (Additional file 1: Table S2).

**Table 4:** Hospitalization rates attributable to influenza and RSV according to risk status and age in the US, 1997–2009 (respiratory broad outcome, any mention)

Age	Rate^a^ of influenza hospitalizations (SD, range)									Rate^a^ of RSV hospitalizations (SD, range)		
	A/H1N1		A/H3N2		B		Total			Total		
	Low risk	High risk	Low risk	High risk	Low risk	High risk	Low risk	High risk	Ratio high/ low risk	Low risk	High risk	Ratio high/low risk
0-4 years	8 (9, 0-23)	0 (0, 0-0)	61 (43, 1-124)	11 (9, 0-26)	41 (29, 2-86)	10 (8, 0-24)	110 (30, 39-147)	21 (10, 5-42)	0.2	526 (90, 431-706)	180 (82, 107-364)	0.3
5-17 years	3 (3, 0-9)	3 (3, 0-9)	8 (6, 0-17)	4 (3, 0-8)	8 (6, 0-15)	3 (3, 0-7)	19 (5, 11-29)	10 (4, 6-16)	0.6	0 (0, 0-0)	0 (0, 0-0)	0.0
18-49 years	1 (1, 0-3)	9 (11, 0-30)	10 (7, 0-20)	52 (35, 1-110)	6 (4, 0-11)	30 (21, 1-64)	17 (5, 6-25)	91 (27, 41-145)	5.5	3 (0, 2-4)	8 (1, 6-10)	2.7
50-64 years	1 (1, 0-1)	10 (12, 0-33)	17 (11, 0-35)	149 (100, 3-313)	7 (5, 0-15)	60 (43, 2-134)	25 (9, 7-37)	220 (82, 76-358)	8.9	5 (0, 5-6)	52 (8, 40-70)	9.8
65-74 years	0 (0, 0-0)	20 (25, 0-81)	39 (26, 1-80)	286 (192, 6-591)	9 (6, 0-18)	44 (32, 1-105)	49 (23, 8-80)	350 (163, 82-594)	7.2	9 (1, 7-11)	104 (17, 78-139)	11.1
75+ years	0 (0, 0-0)	21 (27, 0-86)	117 (80, 3-235)	587 (388, 12-1198)	40 (28, 1-80)	161 (116, 4-380)	157 (68, 29-238)	769 (331, 205-1209)	4.9	81 (11, 65-100)	404 (61, 329-553)	5.0
All ages	2 (2, 0-5)	12 (14, 0-42)	19 (13, 0-39)	176 (117, 4-362)	11 (7, 0-20)	54 (39, 2-124)	32 (9, 11-44)	242 (96, 74-374)	7.7	49 (8, 40-65)	91 (13, 71-119)	1.9

*SD* interseasonal standard deviation, *RSV* respiratory syncytial virus

^a^Annual mean rate per 100,000 population

Overall, high risk versus low risk status was also associated with a higher rate of RSV-attributable hospitalization, with a rate ratio of 1.9 (Table 4). As observed for influenza-attributable hospitalization, the pattern for RSV was inconsistent across age groups. Children up to 18 years of age with high risk status had a lower RSV hospitalization rate than children with low risk status (rate ratio below 1) (Table 4). In adults, RSV-attributable hospitalization rate increased with age in high risk individuals relative to low risk individuals up to age 65–74 years (rate ratio 11.1), followed by a decline to 5.0 in age 75+ years. A similar pattern was observed for the number of hospitalizations (Additional file 1: Table S2).

## Discussion

In our study, we applied a multiple regression modeling strategy that has long been employed to estimate the seasonal excess in hospitalizations attributable to influenza and RSV. We also tested the effect of using a variety of diagnoses outcomes (with varying sensitivity and specificity) beyond the traditional ICD code ranges of cardiac and respiratory diseases. In addition, we examined pediatric age groups more closely and sought to stratify hospitalization by influenza risk status (e.g. mention of certain chronic underlying illnesses). Using an expanded respiratory disease outcome (respiratory broad) that included respiratory signs/symptoms and other viral diseases, we estimated that an average US winter season has ~300,000 influenza-related hospitalization events. The risk of hospitalization for influenza was most pronounced in young children and older adults, with the highest burden occurring in the oldest age group, age 75+ years. The annual relative impact of influenza A and B varied substantially during the study period. Using the same respiratory broad outcome, the model also attributed 200,000 winter-seasonal hospitalizations to RSV, also mostly in young children and older adults. Our results confirm and expand on the findings of a recent CDC study regarding the burden of influenza- and RSV-related hospitalizations in the US [2].

In line with what has been reported in other studies, seasons dominated by circulation of A/H3N2 viruses recorded the highest numbers of influenza-related hospitalizations [1, 2]. The A/H3N2 virus was associated with the majority of influenza-related hospitalizations during our study period. However, we noted that an unusually high 56–74% of influenza hospitalizations were attributable to influenza B during seasons 2000/01, 2002/03, and 2008/09 in which influenza B accounted for 34–47% of all circulating influenza viruses [24]. This implies that simply considering average burden estimates over time misses the fact that an influenza B-dominated season can be associated with considerable influenza burden. This point is particularly important for pediatric age groups for whom the influenza hospitalization burden was as high in a predominantly influenza B season as in any season dominated by influenza A/H3N2 viruses. These excess hospitalizations mostly occurred among children without known underlying medical conditions listed on their hospital discharge records. This finding is consistent with current US and WHO policies of recommending vaccination for children for influenza regardless of risk status, in addition to the traditional recommendations of high risk and senior populations [25, 26].

Our statistical modelling approach included time series of influenza and RSV laboratory-confirmed patterns and thus allowed the simultaneous attribution of hospitalizations to each influenza type/subtype and to RSV. This statistical “excess hospitalizations” approach using national time series of hospitalizations has only in the last decade or so been expanded to include RSV. Recently, evidence of RSV burden was reported by two prospective US studies of laboratory-confirmed influenza and RSV which demonstrated a similar (1:1) ratio of hospitalizations attributable to influenza and RSV in seniors [9, 10]. However, both of those studies were set in local populations (and over fewer seasons) and therefore a national estimate that also allowed computation of the absolute hospital burden was needed. For the entire study period (1997–2009), we found that the average burden of RSV-attributable hospitalizations is indeed substantial, and that its magnitude is nearly half that of influenza in adults aged 65 years and older, and particularly high in those aged 75+ years. Our findings on RSV burden are in excellent agreement with those of Zhou and colleagues [2], as both studies found a substantial RSV burden in seniors but not as high as that of influenza in an average season. As seniors are rarely tested for RSV, additional data on indirect measures of the RSV burden in seniors are needed to guide RSV vaccine development and set recommendations for the RSV vaccines that are expected in the coming years.

Not surprisingly, in children under 5 years of age, the hospitalization burden of RSV was four times higher than that of influenza (ratio 4:1). We found very low RSV-related hospitalization in children aged 5–17 years, as observed in previous studies [27, 28]. As for influenza, most pediatric RSV hospitalizations occurred in children without a documented risk condition for influenza; however, we could not investigate known important risk factors such as premature birth and birth month [29]. Our findings regarding the relative burden of RSV-and influenza-attributable hospitalization across overlapping age groups are also in agreement with other modeling studies [2, 8], but ours is the only study to investigate the RSV burden both in school children and in older seniors aged 75+ years. A caveat to our data is that most RSV hospitalizations occur in children less than 1 year of age [30], but we did not stratify the <5 years age group to give more age-specific estimates. Stratification of this age group would also be useful for influenza estimates.

We noted that the relative burden of influenza B versus A was greatest in both pediatric age groups 0–4 and 5–17 years of age; for these age groups, influenza hospitalizations were split approximately equally between influenza B and A. Overall, the number of hospitalizations was low in the 5–17 year age group regardless of influenza type. Zhou and colleagues also found higher rates of hospitalization attributable to influenza B than to influenza A in children 1–4 years of age, but did not study children aged 5–17 years as a separate age group [2]. In individuals 5–49 years of years, Zhou and colleagues found approximately equal rates of hospitalization attributable to influenza A and B [2].

An important goal of our study was to generate a national estimate of influenza- and RSV-attributable hospitalizations according to medical risk status across all age segments; this aspect was not evaluated in previous studies of US hospitalization burden [1, 2]. Influenza vaccine recommendations around the world account for medical risk status based on the presence of co-morbid conditions. We found our findings in children to be surprising. Although an elevated risk of severe health outcomes in high versus low risk children with influenza is well established in the observational literature [31, 32], we observed the opposite – high risk children had lower hospitalization rates than low risk children. The relative risk of influenza hospitalization in high risk versus low risk children (ratio high risk/low risk) was 0.2 for children 0–4 years of age and 0.6 for those 5–17 years of age. A similar observation has been made by Cromer and colleagues for children 0–4 years of age in an analysis of influenza burden in England, with a relative risk of 0.7 for children <6 months of age and 0.9 for those aged 6 months to 4 years [33]. However, Cromer and colleagues’ results contrast with ours for older children aged 5–17 years; in their study, high risk children had a higher burden of influenza hospitalization than low risk children (relative risk 5.7) [33]. In our previous study of influenza- and RSV-related mortality, we also observed higher death rates in low versus high risk children aged 0–4 years, and approximately equal death rates in low and high risk children aged 5–17 years [21]. There is no obvious explanation for this finding, but it may be related to under-identification and/or under-reporting of risk factors in children, particularly in ICD-coded hospitalization data.

Our study demonstrated the importance of the outcome used to estimate the hospital burden. The traditional P&I and respiratory outcomes are very specific in capturing the influenza-attributable burden, but are not particularly sensitive. In contrast, the classical cardiorespiratory outcome used by Zhou and colleagues [2] has, by definition, a lower specificity due to the inclusion of many cardiac disease events not related to influenza. Thus, estimates of burden using the P&I and respiratory outcomes will be lower than estimates using the cardiorespiratory outcome. The respiratory broad outcome included any mention of respiratory codes, as well as any mention of respiratory signs/symptoms and unspecified virus illness. The inspiration to add codes outside the typical ICD code range used in classical studies came from a study finding that ICD9 code 079.99 (viral disease, unspecified) was the code most often associated with oseltamivir prescription, despite the fact that suspicion of influenza must have prompted the prescription [34]. This finding suggests that likely influenza-related admissions would often be missed using the traditional code range of ICD9 460–519 for respiratory diseases. In our study, as expected, influenza-attributable hospitalizations increased as outcomes became more sensitive and less specific (in rank order P&I, classic respiratory, respiratory broad, cardiorespiratory). We suggest that the respiratory broad outcome provides a case definition that is both sensitive and specific, and thus represents a better choice than traditional outcomes.

To further investigate the potential for exclusion of important hospitalization events using classical outcomes, we also applied the model to time series of sepsis and found that approximately 24,000 sepsis hospitalizations in an average season were attributable to influenza or RSV. Even though this is not a high number compared with the joint seasonal burden of 500,000 hospitalizations associated with the two viruses, the economic burden of sepsis is considerable [35]. This suggests that such outcomes should also be counted and that considerable cost-savings could be made assuming that some sepsis hospitalizations could be prevented by vaccination against influenza or RSV. In principle, such estimations can be done for other disease outcomes with winter-seasonal patterns.

When comparing our hospitalization rates with those of Zhou and colleagues [2], we see multiple differences in outcome definitions, statistical modeling approach (negative binomial versus linear regression), data source (we used the Agency for Healthcare Research and Quality’s NIS database, while Zhou and colleagues used the State Inpatient Databases and projected rates to non-participating States), and use of secondary as well as primary diagnoses in our study. Using primary mention of the classical cardiorespiratory outcome definition, Zhou and colleagues estimated an annual influenza-attributable hospitalization rate of 64 per 100,000 population [2]. Using the corresponding outcome definition, our estimates were slightly higher at 80 per 100,000 population. In an earlier CDC study, Thompson and colleagues reported a corresponding estimate of 88 per 100,000 [1]. For RSV, the hospitalization rate based on primary mention of the cardiorespiratory outcome was estimated at 55 per 100,000 by Zhou and colleagues [2] and at 49 per 100,000 by our analysis. Thus, there is broad agreement on the overall burden and pattern of influenza and RSV disease when similar comparisons are made.

Our study had several limitations. Firstly, hospitalization data were only available by month for non-governmental researchers, whilst virus surveillance data were available by week. We dealt with this by interpolating monthly data into weekly data. It is possible that this approach may have overestimated the precision of hospitalization rates. In addition, the lack of weekly data may have resulted in the model being unable to discriminate precisely the burden of influenza and RSV in seasons where there was significant overlap of the two epidemics, especially in children where viral coinfections (including with respiratory viruses other than RSV and influenza) are common. Secondly, although the relative distribution of influenza A, influenza B, and RSV is known to vary by age, US age-specific virological data were not available; instead, we used all-age composite virological data to guide the timing of the epidemic in the model. However, in sensitivity analyses (not shown), lagging outcomes by age strata relative to virus circulation did not improve model fit, suggesting that the timing at least was adequate, although the relative sizes of the estimates by age group might be affected. Thirdly, risk status could only be determined by the presence or absence of ICD codes during a single hospitalization episode, and assignment of risk status critically depended upon whether the physician had mentioned among the discharge diagnoses any existing underlying disease. In clinical practice, it is likely that the physician would fail to mention some underlying diseases or factors that are categorized as high risk; indeed some important risk factors such as obesity or pregnancy are not reported reliably unless assessed prospectively. This may have led to mis-assignment of risk status. In contrast, symptoms that were part of the influenza illness may have been listed as underlying disease, leading to overestimation of risk. We could not investigate other known risk factors such as premature birth and obesity, which were unlikely to be mentioned by physicians on discharge. Fourthly, negative burden estimates were not included in the aggregations as they are not biologically plausible. In cases where the coefficient indicates a very small attributable burden from one of the viral terms, the estimate may be negative due to the inherent lack of precision. This problem is particularly acute during periods in which RSV and influenza co-circulate, and for nonspecific outcomes such as cardiorespiratory illness. Setting the negative estimates to zero could have resulted in overestimation of hospitalizations. However, we conducted a sensitivity analysis comparing estimates obtained when we did or did not set negative values to zero. For influenza A/H3N2 and B, there were no negative estimates to set to zero. For A/H1N1, where the burden is considerably smaller, we found that setting the estimates to zero did have a substantial effect. However, the net effect on the total influenza burden was limited, because the A/H1N1 virus causes only a small proportion of the total influenza-attributable hospitalization. Finally, although our study used national data and employed a linear regression model, it was limited by the ICD-coded information on each discharge record. Thus, some true patients may have been missed, while others may have been miscoded and wrongly included. It should be noted that the clinical manifestations of RSV infection are very broad, and that atypical presentation of influenza is common in young children; some of these presentations would have been likely to be assigned ICD codes not included in any of our definitions. Moreover, ICD coding practices and criteria for hospitalization can change over time and thus affect the estimates. For example, diagnosis codes in administrative data are often assigned as part of billing and cost reimbursement procedures, allowing changing financial incentives to bias trends in coding practices. It seems unlikely that such forces will become less important in the future, although improvements in diagnostic testing might make the codes assigned to patients with infectious diseases more accurate. No system will allow complete accuracy in assignment of diagnostic codes. Although the two US laboratory-based studies on RSV burden did not have the problem of assignment of diagnostic codes to contend with, they were limited by the use of local data, the small populations under study and the possibility that a hospitalization was not directly attributable to RSV infection [9, 10].

There are also some caveats to our statistical methodology. All indirect statistical approaches based on retrospective data have inherent limitations. For example, non-infectious factors and pathogens other than influenza and RSV may influence hospitalization, but specific information on the nature of such influences is unavailable. Although a cyclic term was included in the model to adjust for this, it adds uncertainty to the model estimates in addition to that derived from variability in the data. In addition, when an outcome occurs at a low frequency, the assumption of a normally distributed dependent variable is more likely to be violated. To address the problems associated with low frequency data, some previous analyses have used generalized linear models that relax the assumption of a normal distribution, including discrete probability distributions appropriate for count data such as the Poisson regression with a natural logarithm for the link function [12, 36]. Use of the logarithm function, however, implies multiplicative effects of respiratory viruses, which is an unrealistic assumption [37, 38]. It is more likely that co-circulating strains could result in a reduction of the burden due to cross-protection [39], competitive exclusion or both. This makes the additive model more plausible from a biological and epidemiological perspective [38–40].

## Conclusions

This is the first study to model estimates of influenza- and RSV-attributable hospitalizations in the US according to a novel outcome (respiratory broad), and to use expanded age groups and risk based status across all age groups. Our findings are in line with and expand upon other studies that have found a high burden of influenza- and RSV-attributable hospitalization in children, older adults, and at-risk groups, and add to the growing evidence for RSV as an important pathogen in adult hospitalization. We confirmed that seasonal RSV and influenza hospitalization burden has a U-shaped age-related pattern, with most hospitalizations occurring in low risk children and high risk older adults. The study demonstrated the importance of the outcome used to estimate hospitalization burden, and it study adds to the body of evidence that informs public health policies on vaccination strategies to control influenza and RSV.

## Additional file

Additional file 1: Table S1. Seasonal burden of hospitalization attributable to influenza and RSV by season in the US, 1997–2009 (respiratory broad outcome, any mention). ^1^Annual mean rate per 100,000 population; *Data included up to 31st March 2009; CI: confidential interval. **Table S2.** Number of hospitalizations attributable to influenza and RSV according to risk status and age in the US, 1997–2009 (respiratory broad outcome, any mention). SD: standard deviation; RSV: respiratory syncytial virus. (DOCX 42 kb)

## Abbreviations

CDC: Centers for disease control and prevention
ICD: International classification of disease
NHIS: National health information survey
NIS: Nationwide inpatient sample
NREVSS: National respiratory and enteric virus surveillance system
RSV: Respiratory syncytial virus
WHO: World Health Organization
