# Supplementary material for: Estimates of hospitalization attributable to influenza and RSV in the US during 1997–2009, by age and risk status
Source: BMC Public Health. 2017 Mar 21;17:271. doi: 10.1186/s12889-017-4177-z (PMC5359836; doi:10.1186/s12889-017-4177-z)
Supplement: Additional file 1: Table S1. — Seasonal burden of hospitalization attributable to influenza and RSV by season in the US, 1997–2009 (respiratory broad outcome, any mention). 1Annual mean rate per 100,000 population; *Data included up to 31st March 2009; CI: confidential interval. Table S2. Number of hospitalizations attributable to influenza and RSV according to risk status and age in the US, 1997–2009 (respiratory broad outcome, any mention). SD: standard deviation; RSV: respiratory syncytial virus. (DOCX 42 kb) [file 12889_2017_4177_MOESM1_ESM.docx]

# Supporting information

**S1 table. Seasonal burden of hospitalization attributable to influenza and RSV by season in the US, 1997-2009 (respiratory broad outcome, any mention)**

**S2 table. Number of hospitalizations attributable to influenza and RSV according to risk status and age in the US, 1997-2009 (respiratory broad outcome, any mention)**

S1 table. Seasonal burden of hospitalization attributable to influenza and RSV by season in the US, 1997-2009 (respiratory broad outcome, any mention)

|  | **Influenza** | | | | | | | | **RSV** | |
| --- | --- | --- | --- | --- | --- | --- | --- | --- | --- | --- |
|  | **A/H1N1** | | **A/H3N2** | | **B** | | **Total** | | **Total** | |
|  | **Number (95% CI)** | **Rate^1^ (95% CI)** | **Number (95% CI)** | **Rate^1^ (95% CI)** | **Number (95% CI)** | **Rate^1^ (95% CI)** | **Number** | **Rate^1^** | **Number (95% CI)** | **Rate^1^ (95% CI)** |
| 1997/98 | 372  (364-380) | 0.14 (0.13-0.14) | 237085 (236151-238019) | 87.73 (87.38-88.07) | 2882 (2850-2915) | 1.07 (1.05-1.08) | 240339 | 88.94 | 218476 (216448-220504) | 80.84 (80.09-81.59) |
| 1998/99 | 938 (911-966) | 0.34 (0.33-0.35) | 220555 (219726-221384) | 80.88 (80.58-81.18) | 99778 (98818-100737) | 36.59 (36.24-36.94) | 321271 | 117.81 | 192254 (190377-194131) | 70.50 (69.81-71.19) |
| 1999/2000 | 3806 (3732-3880) | 1.35 (1.32-1.38) | 258300 (257309-259292) | 91.54 (91.19-91.89) | 2911 (2880-2942) | 1.03 (1.02-1.04) | 265017 | 93.92 | 210436 (208329-212543) | 74.58 (73.83-75.33) |
| 2000/01 | 51051 (49939-52163) | 17.91 (17.52-18.30) | 3750 (3735-3765) | 1.32 (1.31-1.32) | 155753 (154226-157281) | 54.66 (54.12-55.19) | 210554 | 73.89 | 183071 (181215-184927) | 64.24 (63.59-64.89) |
| 2001/02 | 3392 (3324-3460) | 1.18 (1.16-1.20) | 276471 (275479-277463) | 96.12 (95.78-96.47) | 65985 (65365-66606) | 22.94 (22.73-23.16) | 345848 | 120.24 | 195617 (193762-197473) | 68.01 (67.37-68.66) |
| 2002/03 | 53022 (52000-54045) | 18.28 (17.92-18.63) | 29142 (28996-29288) | 10.05 (10.00-10.10) | 154639 (153082-156195) | 53.30 (52.77-53.84) | 236803 | 81.63 | 164153 (162509-165798) | 56.58 (56.02-57.15) |
| 2003/04 | 49 (48-50) | 0.02 (0.02-0.02) | 392352 (391009-393696) | 134.00 (133.54-134.46) | 5949 (5882-6016) | 2.03 (2.01-2.05) | 398350 | 136.05 | 171978 (170245-173712) | 58.73 (58.14-59.33) |
| 2004/05 | 410 (402-418) | 0.14 (0.14-0.14) | 256815 (255936-257693) | 86.90 (86.61-87.20) | 123802 (122693-124911) | 41.89 (41.52-42.27) | 381027 | 128.93 | 151326 (149803-152850) | 51.21 (50.69-51.72) |
| 2005/06 | 11094 (10834-11353) | 3.72 (3.63-3.80) | 217435 (216714-218156) | 72.87 (72.63-73.11) | 94124 (93297-94952) | 31.55 (31.27-31.82) | 322653 | 108.14 | 161895 (160231-163560) | 54.26 (53.70-54.82) |
| 2006/07 | 77994 (76294-79695) | 25.89 (25.33-26.46) | 71766 (71495-72036) | 23.82 (23.73-23.91) | 102850 (101884-103816) | 34.14 (33.82-34.46) | 252610 | 83.85 | 190328 (188447-192209) | 63.18 (62.56-63.81) |
| 2007/08 | 37049 (36388-37709) | 12.18 (11.97-12.40) | 247669 (246834-248505) | 81.45 (81.17-81.72) | 188309 (186598-190020) | 61.92 (61.36-62.49) | 473027 | 155.55 | 187315 (185451-189178) | 61.60 (60.98-62.21) |
| 2008/09* | 46154 (45286-47023) | 15.11 (14.83-15.40) | 8602 (8570-8633) | 2.82 (2.81-2.83) | 68321 (67689-68954) | 22.37 (22.16-22.58) | 123077 | 40.30 | 207015 (204891-209138) | 67.78 (67.08-68.47) |
| Average season | 23778 | 8.02 | 184995 | 64.13 | 88775 | 30.29 | 297548 | 102.44 | 186155 | 64.29 |

^1^ Annual mean rate per 100,000 population

*Data included up to 31st March 2009

CI: confidential interval

CIs determined for the regional estimates were aggregated at a national level by pooling the variances of the regional estimates (σ^2^_region_) under the assumption that estimates were independent across regions (, i.e. σ_ij_ = 0 $\sigma_{\mathrm{ij}}=0$for regions i and j ) and their variances homogeneous:

σ^2^_national_ = Σσ^2^_region_

CI_national_ = α_national_ ± 1.96 * √σ^2^_national_

S2 table. Number of hospitalizations attributable to influenza and RSV according to risk status and age in the US, 1997-2009 (respiratory broad outcome, any mention)

| **Age** | **Number influenza hospitalizations (SD, range)** | | | | | | | | | **Number RSV hospitalizations  (SD, range)** | | |
| --- | --- | --- | --- | --- | --- | --- | --- | --- | --- | --- | --- | --- |
|  | **A/H1N1** | | **A/H3N2** | | **B** | | **Total** | | | **Total** | | |
|  | **Low risk** | **High risk** | **Low risk** | **High risk** | **Low risk** | **High risk** | **Low risk** | **High risk** | **Ratio high/ low risk** | **Low risk** | **High risk** | **Ratio high/ low risk** |
| 0-4 years | 1365 (1543, 5-4165) | 0 (0, 0-0) | 10799 (7541, 218-22166) | 208 (166, 1-474) | 7283 (5190, 289-15241) | 180 (145, 4-434) | 19448 (5226, 7216-25165) | 388 (167, 102-740) | 0.02 | 93129 (13947, 77554-121619) | 3316 (1386, 2102-6456) | 0.04 |
| 5-17 years | 1271 (1493, 2-4144) | 271 (315, 0-812) | 3681 (2522, 76-7749) | 356 (247, 5-706) | 3507 (2459, 130-6798) | 292 (216, 6-579) | 8459 (2310, 4840-12953) | 919 (330, 554-1508) | 0.11 | 100 (13, 78-119) | 0 (0, 0-0) | 0.00 |
| 18-49 years | 1150 (1303, 2-3179) | 2480 (2938, 5-8704) | 10414 (7065, 216-21682) | 13666 (9384, 261-29823) | 6130 (4297, 220-11902) | 8082 (5791, 224-18580) | 17694 (5211, 6855-26641) | 24228 (8067, 12127-42364) | 1.37 | 3070 (358, 2533-3752) | 2093 (385, 1694-3063) | 0.68 |
| 50-64 years | 163 (188, 0-452) | 2023 (2401, 2-7000) | 4907 (3322, 95-10581) | 26514 (18497, 482-58058) | 2237 (1595, 64-5061) | 11521 (8697, 266-29935) | 7306 (2971, 2362-12460) | 40058 (18242, 17317-79641) | 5.48 | 1576 (205, 1354-2060) | 9517 (2795, 7048-16014) | 6.04 |
| 65-74 years | 0 (0, 0-0) | 2157 (2777, 2-9005) | 3408 (2280, 73-6717) | 29167 (19460, 568-60415) | 787 (551, 27-1602) | 4642 (3501, 101-12256) | 4195 (2019, 745-6765) | 35966 (17032, 9875-60707) | 8.57 | 815 (116, 628-993) | 10850 (2326, 8134-16590) | 13.31 |
| 75+ years | 0 (0, 0-0) | 2377 (3093, 2-10099) | 7657 (5294, 172-15374) | 62875 (41640, 1264-131654) | 2603 (1856, 95-5332) | 17817 (13198, 415-45284) | 10260 (4468, 1837-15547) | 83069 (37297, 24607-140286) | 8.10 | 5279 (819, 4242-6686) | 43947 (8926, 36650-66450) | 8.32 |
| **All ages** | **3949 (4461, 10-10670)** | **9309 (11397, 11-35620)** | **40866 (27861, 849-84269)** | **132787 (89029, 2581-280822)** | **22547 (15794, 831-43043)** | **42533 (31356, 1022-106923)** | **67362 (19889, 23856-95233)** | **184629 (78489, 64582-323600)** | **2.74** | **103969 (14921, 86504-134109)** | **69723 (13783, 56808-104220)** | **0.67** |

SD: standard deviation; RSV: respiratory syncytial virus
